# The Early Evolutionary History of Belemnites: New Data from Japan

**DOI:** 10.1371/journal.pone.0095632

**Published:** 2014-05-02

**Authors:** Yasuhiro Iba, Shin-ichi Sano, Jörg Mutterlose

**Affiliations:** 1 Department of Natural History Sciences, Hokkaido University, Sapporo, Japan; 2 Fukui Prefectural Dinosaur Museum, Katsuyama, Japan; 3 Institute of Geology, Mineralogy and Geophysics, Ruhr-University Bochum, Bochum, Germany; Northern Illinois University, Canada

## Abstract

Belemnites (Order Belemnitida), a very successful group of Mesozoic coleoid cephalopods, dominated the world's oceans throughout the Jurassic and Cretaceous. According to the current view, the phylogenetically earliest belemnites are known from the lowermost Jurassic (Hettangian, 201–199 Ma) of northern Europe. They are of low diversity and have small sized rostra without clear grooves. Their distribution is restricted to this area until the Pliensbachian (191–183 Ma). Here we describe two new belemnite taxa of the Suborder Belemnitina from the Sinemurian (199–191 Ma) of Japan: *Nipponoteuthis katana* gen et sp. nov., which represents the new family Nipponoteuthidae, and *Eocylindroteuthis* (?) *yokoyamai* sp. nov. This is the first reliable report of Sinemurian belemnites outside of Europe and the earliest record of typical forms of Belemnitina in the world. The Sinemurian belemnites from Japan have small to large rostra with one deep and long apical groove. Morphologically these forms are completely different from coeval European genera of Hettangian–Sinemurian age. These new findings suggest that three groups of Belemnitina existed in the Hettangian–Sinemurian: 1) European small forms, 2) Japanese very large forms, and 3) the typical forms with a distinctive apical groove, reported here. The Suborder Belemnitina therefore did not necessarily originate in the Hettangian of northern Europe. The new material from Japan documents that the suborder Belemnitina had a much higher diversity in the early Jurassic than previously thought, and it also shows strong endemisms from the Sinemurian onwards.

## Introduction

Belemnites (Order Belemnitida), which became extinct at the Cretaceous/Paleogene boundary, were extremely abundant in Jurassic and Cretaceous oceans. Their diversity shifts are closely correlated to environmental changes, including extinction events and climate variations. An understanding of the causes and mechanisms behind the radiation of belemnites provides clues to the evolutionary dynamics of Mesozoic nektonic biota [1]–[3]. Although the evolutionary history of belemnites during the late Early Jurassic and Cretaceous has been well studied [1]–[7], their early evolution is poorly understood. According to the current view, belemnites originated in the earliest Jurassic (Hettangian, 201–197 Ma) in northern Europe as very small forms (*Schwegleria*). Their paleobiogeographic distribution was restricted to northern Europe and the Mediterranean area (e.g., Turkey) until the Pliensbachian (191–183 Ma) [8].

Recently two belemnite taxa have been described from the Hettangian of Japan, which are completely different from the Early Jurassic belemnites of Europe [9]. These authors concluded that belemnites originated in the Late Triassic ∼33 million years earlier than previously thought (Carnian, 237–228 Ma) outside Europe. The morphological differences between the Hettangian belemnites from Japan and those from Europe and elsewhere are considerable, but their systematic relationship remains unsolved. A better understanding of the early evolution of belemnites may therefore be provided by new material from the post-Hettangian Lower Jurassic of East Asia.

Here, we describe belemnites of Sinemurian age from outside Europe for the first time. The new species from Japan have small–large rostra with one deep and long apical groove. These forms are totally different from the small rostra of the coeval belemnite assemblages from Europe, which are missing prominent grooves. The distinctive features of the Japanese belemnites clearly indicate that they can safely be assigned to the Suborder Belemnitina, the dominant group of belemnites during the earliest Jurassic to Early Cretaceous. We provide here a better understanding of the early evolutionary history of the Belemnitida.

### Geological Settings and Materials

**Figure 1:**
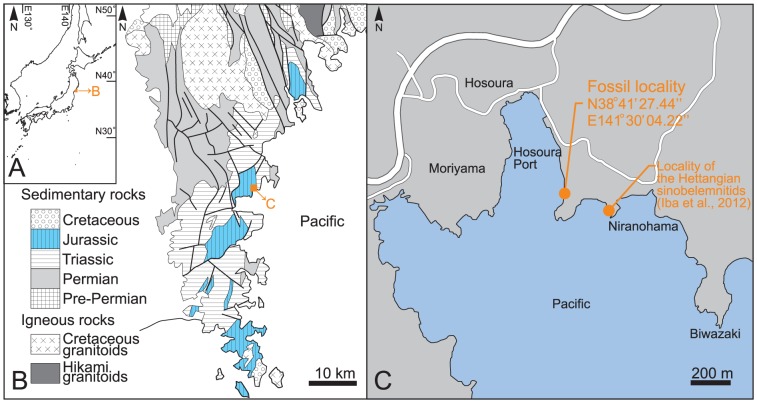
Locality map with geological information [29] for Sinemurian belemnites in northeastern Japan. The locality where the Hettangian sinobelemnitid and the very large form of the Belemnitina were found [9] is also indicated.

Forty-two identifiable specimens (UHR 33222–33263) of belemnites were recovered from the Sinemurian of the Shizukawa Group, which crops out along the Pacific coast of northeastern Japan (Figure. 1). This lithostratigraphic unit consists of the Niranohama (middle–upper Hettangian) and the Hosoura (Sinemurian–Aalenian) formations [10], [11]. Recently, *Sichuanobelus utatsuensis* and a very large form of the Belemnitina (Belemnitina fam., gen. et sp. indet) have been described from the Niranohama Formation [9]. The Hosoura Formation is subdivided into four lithological units; Hi, Ha, Hl, and Hh in ascending order [10]. The abundant belemnites including the two Belemnitina species described here, and *Sichuanobelus* sp. (familiy Sinobelemnitidae) have been collected from sandstones with mud matrix in the upper part of the Unit Ha (Figure 1). This sandstone often contains pebbly lag deposits, where plant fossils, ammonites (*Arnioceras*), and belemnites have been found. Since almost all calcitic rostra are weathered and preserved as external molds, they were recovered as silicone rubber casts. Phragmocones and protoconchs that are usually filled with sediments and/or calcite have rarely been discovered. The unit Ha contains age diagnostic ammonites (e.g., *Arnioceras yokoyamai*) that clearly indicate a Sinemurian age [12]. All specimens in this study are deposited in the collections of the University Museum, Hokkaido University, Japan (UHR).

## Results

### Systematic Paleontology

#### Order Belemnitida Zittel, 1895 [13]

Remarks: The two belemnite species described here can be clearly distinguished from the Order Aulacocerida by showing the diagnostic features of the Belemnitida: (1) a short alveolar part of the rostrum with a high apical angle of the phragmocone (>25 degree), and (2) narrow concentric growth lines in the transverse section of the rostrum, indicating originally calcitic ones.

#### Suborder Belemnitina Zittel, 1895 [13]

Remarks: The absence of alveolar grooves and the presence of apical grooves on the rostrum are diagnostic features of the Belemnitina.

#### Family Nipponoteuthidae, new family

urn:lsid:zoobank.org:act:5E3EDFAE-9905-4145-9689-7B24CFA66D0C

Type genus: *Nipponoteuthis* gen. nov.

Etymology: Nippon is the Japanese name for Japan, teuthis is squid in Greek.

Diagnosis: Rostrum strongly laterally compressed; Single prominent apical groove on the presumably ventral side; Outline symmetrical; Profile asymmetrical and kayak-shaped; Apical region very sharp with a needle-like apex; Alveolar region extremely short (less than one twenty-fifth of rostrum length).

Remarks: The family Nipponoteuthidae differs from all other belemnite families by its unique morphology showing a kayak-shaped profile, a strongly laterally compressed rostrum, a needle-like apex, an extremely short alveolar region, and a long, deep apical groove.

### Genus *Nipponoteuthis* n. gen

urn:lsid:zoobank.org:act:17E90180-8ECD-4E6D-946B-53AFED55BB5A

Type species: *Nipponoteuthis katana* sp. nov.

Diagnosis: Rostrum small to medium size (33–76 mm) with long and deep apical groove, laterally compressed; Outline symmetrical; Profile asymmetrical and kayak-shaped; Apical region laterally thin, very sharp with needle-like apex; Anterior end of rostrum circular in transverse section; Alveolar region extremely short; Phragmocone penetrates less than one twenty-fifth of the rostrum; Apical groove long and v-shaped; Two weak lateral lines on apical–stem region; No epirostrum, nor alveolar grooves or striae.

Comparison: Very slender rostra of *Nipponoteuthis* slightly resemble those of *Salpingoteuthis* (Lower Toarcian to Aalenian of Europe), *Youngibelus* (Lower Toarcian of Europe), and *Bairstowius* (Upper Sinemurian to Pliensbachian of Europe and Turkey) [14], [15]. *Nipponoteuthis* can be clearly distinguished from these three genera by having an extremely short alveolar region, a deeper apical groove, and a kayak-shaped rostrum with a needle-like apex. *Salpingoteuthis* possesses multiple ventral and dorsal apical grooves, and a symmetrical profile [14]. *Youngibelus* is marked by laterally moderately compressed rostra without apical grooves [14]. *Bairstowius* has triple longitudinal lateral furrows of Hastites like aspect; the apex is commonly striated [15]. These diagnostic features of the three genera are missing in *Nipponoteuthis*.

### Species Nipponoteuthis katana n. sp

urn:lsid:zoobank.org:act:E8F0BE52-9159-4863-AE47-ADA3485FB32F

Materials: 33 specimens (UHR 33222 to 33254)

Holotype: UHR 33222

Paratypes: UHR 33223 to 33227

Type locality and horizon: Ha unit of the Hosoura Formation of the Shizukawa Group on the east shore of the Hosoura port, Minamisanriku Town, Miyagi Prefecture, northeastern Japan.

Etymology: Katana is the Japanese name for “sword”, which is characterized by a thin and long, slightly curved, single-edged and pointed shape.

Diagnosis: As for the genus.

**Figure 2:**
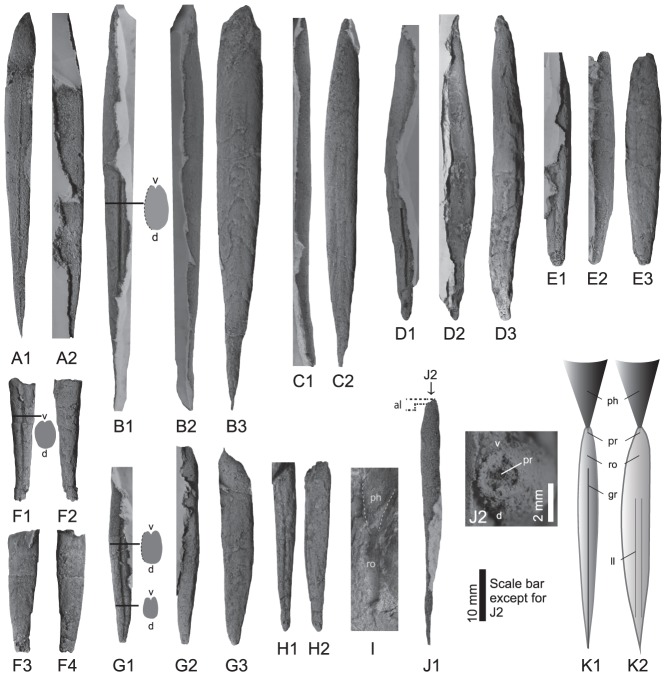
*Nipponoteuthis katana* n. gen., n. sp. from the Sinemurian of northeastern Japan. A: holotype, UHR 33222, possible ventral (A1) and left lateral (venter to the right) (A2). B: paratype, UHR 33223, possible ventral, a transverse section (B1), dorsal (B2), left lateral (B3). C: paratype, UHR 33225, possible dorsal (C1), left lateral (C2). D: UHR 33228, possible ventral (D1), dorsal (D2), left lateral (D3). E: UHR 33229, possible ventral (E1), dorsal (E2), right lateral (E3). F: paratype, UHR 33226, possible ventral, a transverse section (F1), dorsal (F2), right lateral (F3), left lateral (F4). G: UHR 33230, possible ventral, transverse sections (G1), dorsal (G2), left lateral (G3). H: UHR 33231, possible ventral (H1), right lateral (H2). I: UHR 33232, lateral, rostrum with phragmocone. J: paratype, UHR 33227, light lateral (J1), transverse view of extremely short alveolar region from the front side (J2). K: schematic reconstruction of *Nipponoteuthis katana*, possible ventral (K1), left lateral (K2). Prostracum is not indicated. All except I and K are silicone rubber casts from external molds in the outcrop. Abbreviations: v–ventral; d–dorsal; ph–phragmocone; ro–rostrum; al–alveolar region; pr–protoconch, gr–groove, ll–lateral line.

Description: Rostrum small–medium size. Lengths of complete rostra are 33.5 to 76.5 mm. Maximum diameter located in the middle part of the rostrum (Figure 2A–I). Maximum dorso-ventral diameter (Dv) and lateral diameter (Dl) of holotype are 5.9 mm and 4.5 mm, respectively. Dv/Dl ranges from 1.3 to 1.7. Rostrum strongly laterally compressed (especially in small individuals) and lateral sides are slightly rounded (Figure 2B, F, G). Outline is symmetrical (Figure 2A). Profile is asymmetrical and kayak-shaped (side of groove is straight and opposite side is slightly curved) (Figure 2C). Apical region is very sharp with a needle-like apex (Figure 2A, B, J). Anterior end of rostrum also small in diameter and circular in transverse section (Figure 2J). Alveolar region extremely short (Figure 2J, K). Phragmocone penetrates less than one twenty-fifth of the rostrum length. Apical angle of phragmocone is approximately 28° (Figure 2I). Apical groove long; it starts from the apex, and extends over half of the rostrum (Figure 2A). Groove is deep and v-shaped in the transverse section (Figure 2B, F, G). Two lateral lines extend from the apical region to the stem region, but do not reach the alveolar region (Figure 2B, C). Concentrated growth lines at the surface of the apical region indicate that there is no epirostrum developed (Figure 2B, C). No alveolar grooves or striae.

### Family Uncertain

Remarks: The Aalenian–Bajocian genus *Eocylindroteuthis* Riegraf 1980 [16] has been included in the family Cylindroteuthidae [5], [16]–[18]. Recently, it has been pointed out that *Eocylindroteuthis* may be identical or at least closely related to the genus *Homaloteuthis* of the family Megateuthidae [19]. Similarities between *Eocylindroteuthis*, *Homaloteuthis* and *Megateuthis*, especially in their early ontogenetic stages, have been discussed [18]. It is therefore debatable whether *Eocylindroteuthis* belongs to the Cylindroteuthidae or to the Megateuthidae. The family assignment of this genus is therefore considered as uncertain in this paper.

### Genus *Eocylindroteuthis* Riegraf 1980 [16]

Remarks: The specimens from Japan resemble *Eocylindroteuthis* by showing the following features: 1) an intermediate groove possibly on the ventral side, which never reaches the alveolar region, 2) a cylindrical outline, 3) a laterally compressed rostrum, which is therefore elliptical in transverse section. The ventral groove of *Eocylindroteuthis*, however, is shallower than that in the specimens described here from Japan. This feature allows differentiation between the two taxa. The genus *Holcobelus* (Holcobelidae, Belemnitina) from Europe also possesses an intermediate ventral groove that usually reaches the alveolar region [7]. The “*Holcobelus*” [20], described from Siberia, differs from proper *Holcobelus* by having an apical groove or flattening on the dorsal side. None of the features typical for the European *Holcobelus* nor the Russian “*Holcobelus*” can be observed in the Japanese specimens.

The genus *Eocylindroteuthis* first appeared in the Aalenian [18], [19]. A huge stratigraphic gap between the Sinemurian specimens described here from Japan and the Aalenian–Bajocian species from Europe therefore exists. Due to the lack of information about the ontogenetic changes of the rostra, a precise assignment of the present specimens is currently difficult. Additional specimens, with preserved internal structures, are needed for their precise assignment to this or possibly to a new genus. The specimens from Japan are therefore tentatively assigned to *Eocylindroteuthis*.

### Species Eocylindroteuthis (?) yokoyamai n. sp

urn:lsid:zoobank.org:act:20D174C6-5A7E-473A-B6A4-6262369C0FF3

Materials: 9 specimens (UHR 33255 to 33263)

Holotype: UHR 33255

Paratype: UHR 33256

Type locality and horizon: Same as for *Nipponoteuthis katana*.

Etymology: In honor of Professor Matajiro Yokoyama (1860–1942), a Japanese paleontologist who described belemnites from the Shizukawa area (Japan) for the first time in 1904.

Diagnosis: Rostrum small–large size (28–124 mm), cylindrical and laterally compressed; Outline symmetrical; Profile asymmetrical; Intermediate and u-shaped deep groove on the ventral side, which never reaches the alveolar region; Ventral side flattened; Dorsal side rounded; Alveolar region short; Phragmocone penetrates one eighth of the rostrum length.

**Figure 3:**
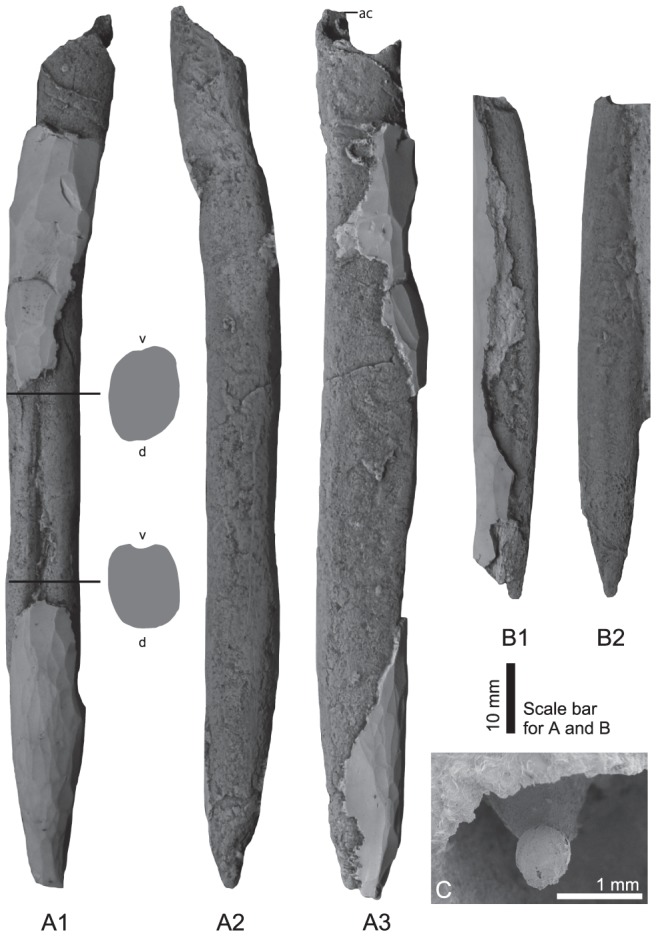
*Eocylindroteuthis* (?) *yokoyamai*, n. sp. from the Sinemurian of northeastern Japan. A: holotype, UHR 33255, ventral, transverse sections (A1), dorsal (A2), left lateral (A3). B: paratype, UHR 33256, ventral (B1), right lateral (B2). C: protoconch in the transverse view from the front side, UHR 33263. All except C are silicone rubber casts from external molds in the outcrop. Abbreviations: v–ventral; d–dorsal. ac–alveolar contact.

Description: The cylindrical rostrum is of small–large size. Lengths of the complete rostra are 28.2 to 124.2 mm. The maximum diameter is located in the middle part of the rostrum (Figure 3A). Maximum dorso-ventral diameter (Dv) and lateral diameter (Dl) of the holotype (largest specimen) are 13.6 mm and 9.7 mm, respectively. Dv/Dl is ∼1.4. The rostrum is laterally compressed with slightly flattened lateral sides. The possible dorsal side (non-groove side) is rounded. The outline is symmetrical. The profile of the apical region is slightly asymmetrical and that of other parts is symmetrical. Apex is moderately acute (Figure 3A). Alveolar region is short and the phragmocone penetrates one eighth of the rostrum length. Apical angle of phragmocone is approximately 25 degree. There is an intermediate and u-shaped groove, which never reaches the alveolar region (Figure 3A, B). Two weak lateral lines of equal shape can be observed in the paratype (UHR 33256) (Figure 3B). The protoconch is oval, 0.77 and 0.65 mm in the major and the minor axis, respectively (Figure 3C).

Comparison: *E*. (?) *yokoyamai* can be distinguished from two well known species of *Eocylindroteuthis*, *E*. *corneliaschmittae* (upper Aalenian–Lower Bajocian of southwestern Germany, northeastern France, Luxembourg, Belgium, possibly northwestern Germany) and *E. trautscholdi* (Lower Bajocian of southwestern Germany, northeastern France, Luxembourg, Belgium, Switzerland) by having a deeper groove and a shorter alveolar region. These latter two species have a ventral flattening instead of a groove and a much longer alveolar region (one third to one quarter, one half to one third of the rostrum, respectively) [18].

## Discussion and Conclusions

**Figure 4:**
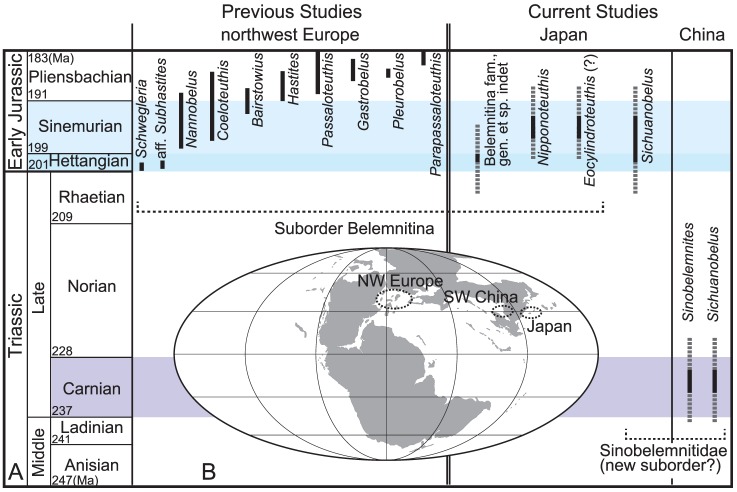
Stratigraphic ranges of belemnite genera from the Triassic to Pliensbachian in Europe, Japan, and China. (A) The paleomap [30] shows the position of northwest Europe, Japan, and southwest China at 200Ma (B). Three important stages, the Carnian, Hettangian, and the Sinemurian are highlighted. The Triassic Sinobelemnitids, the belemnites from the Hettangian of Japan, and the Early Jurassic belemnite occurrences from Europe are based on previous literature [5], [9], [23], [24].

The Order Belemnitida has been subdivided into two suborders based on the presence of alveolar grooves (Belemnopseina [21]  =  Pachybelemnopseina [22]) or its absence (Belemnitina) [21]. Instead of alveolar grooves, the Suborder Belemnitina has apical ones [21]. Generally, the Suborder Belemnopseina has an alveolar groove on the ventral side with the exception of the Duvaliidae, which has it on the dorsal side [3]. Previous hypotheses of the early evolution of belemnites are summarized as follows. Belemnites evolved in Europe as small forms (*Schwegleria*; about 10 mm in length of rostrum) representing the Belemnitina in the Hettangian [5], [8], [23]. Their distribution was restricted to the European shelf seas for 18 million years until the Pliensbachian, they diversified and expanded worldwide in the Toarcian [8]. The Suborder Belemnopseina first appeared in the Middle Jurassic [5]. Recently, lost records of earliest belemnites (the Sinobelemnitidae) have been re-discovered from the Hettangian of Japan and the Carnian (Upper Triassic) of southwest China [9]. The Sinobelemnitidae are characterized by enigmatic morphological features, including the presence of a deep dorsal alveolar groove with a splitting surface and the absence of any apical and/or ventral alveolar grooves [9], [24]. Since such features are generally unknown in the existing two suborders (the Belemnitina and the Belemnopseina), the Sinobelemnitidae may represent a separate group (new suborder) within the Order Belemnitida [9]. These new findings extend the origin of the belemnites back by 33 million years into the Late Triassic (Figure 4). The lines of evidence strongly indicate that previous scenarios of the early evolution of belemnite are no longer up-to date and are biased by the fact that most studies have focused on European records (Figure 4).

The Suborder Belemnitina is the most dominant group of belemnites in the Jurassic to Early Cretaceous oceans. Morphological differences between the Late Triassic–Early Jurassic Sinobelemnitidae and Early Jurassic Belemnitina are distinct, but their systematic relationships remain unresolved. To understand the early evolutionary history of belemnites, the phylogeny, biogeography and diversification of the Belemnitina in the Early Jurassic is one of most important issues to address. So far early Belemnitina (Hettangian–Sinemurian) from Europe include five genera: *Schwegleria*, aff. *Subhastites, Nannobelus*, *Coeloteuthis* and *Bairstowius* [5], [23] (Figure 4). These taxa are characterized by rostra with a very weak and short apical groove or even an absence of grooves [5], [8], [23].

The belemnite genus “*Salpingoteuthis*” was reported from the Sinemurian of southern Tibet [25]. The Tibetan specimen is poorly preserved and does not show any diagnostic characters of *Salpingoteuthis*, such as multiple ventral and dorsal apical grooves. Thus we exclude the Tibetan record from the discussion of this paper. Recently, a very large taxon of the Belemnitina, based on two fragments of the alveolar part of the rostrum, has been reported from the Hettangian of Japan [9]. The assignment of these two fragments to the Belemnitina is based on their very large size and the absence of an alveolar groove. A distinctive apical groove, which is one of the most important diagnostic characters of the Belemnitina, cannot be confirmed for these specimens.

The two new species here described are characterized by small to large rostra with one single deep and long apical groove (Figures 2 and 3). Hence, these forms from Japan differ from the coeval European ones, which have conical and short rostra with very weak or even without apical grooves. These morphological features are diagnostic for the Suborder Belemnitina, and typical for post-Sinemurian taxa of this suborder in Europe. They are unknown from earlier belemnites of Late Triassic to Sinemurian age. The size of the rostra of *E* (?). *yokoyamai* (over 12 cm in length: Figure 3A) by far exceeds that of the Belemnitina from the earliest Jurassic of Europe. It more closely approaches the size of Pliensbachian to Middle Jurassic belemnites from Europe. The record of belemnites from the Sinemurian of Japan is not only the first reliable evidence of the occurrence of Sinemurian Belemnitina from outside Europe, but is also the earliest record of the typical Belemnitina in the world. Phylogenetic relationships between the Japanese Sinemurian forms and the late Early Jurassic European Belemnitina, both of which share the diagnostic character of a distinct apical groove, should attract more attention in order to clarify the early evolution of the Belemnitina in future studies. If the presented data are confirmed, the small European Hettangian–Sinemurian belemnites can be considered as an endemic offshoot of the Belemnitina or possibly even the Belemnitida.

Taking these new findings from Japan into account, three groups of Belemnitina can be differentiated at the moment for the Hettangian–Sinemurian interval. These include 1) European small forms, 2) Japanese very large forms, and 3) forms with a distinctive apical groove, reported here. Therefore, the Suborder Belemnitina did not necessarily originate in the Hettangian of northern Europe and had a higher diversity in the Sinemurian than previously thought (Figure 4). It has been believed that belemnites expanded their geographic distribution worldwide in the Toarcian. At the same time a strong endemism developed, documented by the presence of Tethyan and Boreal belemnite faunas) [8], [18]. Our new findings indicate that the endemic evolution of the belemnite (Belemnitina) faunas was already established in the Sinemurian, 15 million years earlier than previously thought. Further studies of earliest Jurassic and possibly Triassic belemnites outside Europe are necessary to clarify the early evolutionary history of the Belemnitina.

The grooves in the rostra are one of the most important characters of the Belemnitida, and can be used as diagnostic characters dividing the suborders of belemnites [21], [22]. Usually they have been interpreted as an attachment scar for soft tissue such as blood vessels [26]. The difference in the position and numbers of grooves in the rostra and the resulting implications for the soft tissues of the body are not yet fully understood. This study indicates that an apical groove of the Belemnitina had already been established in the Sinemurian. A dorsal alveolar groove was acquired by the Sinobelemnitidae even earlier, in the Carnian (Late Triassic). The earliest records of ventral alveolar grooves are known from *Holcobelus* (Belemnopseina) in the Middle Jurassic [7]. The Dicoelitidae have both dorsal and ventral alveolar grooves on a single rostrum, their earliest record is from the Toarcian of Canada and possibly southern Tibet [27], [28]. These informations provide the background to consider the origin, role and function of different type of grooves in belemnites. This may result in a better understanding of the phylogenic relationships of belemnite suborders, as well as of other coleoid groups.

## Methods and Ethics Statements

A total of 42 identifiable specimens (UHR 33222–33263) of belemnites have been collected June 2012, February and September 2013, at the outcrop in the Hosoura fishing harbor of the Minamisanriku Town, Miyagi Prefecture, northeastern Japan (Figure 1). All calcitic rostra are weathered and preserved as external molds. They were recovered as silicone rubber casts using by Putty-Type of Exafine (GC Co., Ltd. Japan), directly from the outcrop. For observing details of shell ornaments and photographs, all specimens were firstly coated by colloidal graphite, diluted with ethanol, and then recoated by sublimated ammonium chloride. A zoom microscope, Axio Zoom V16 (Carl Zeiss Microscopy, Germany) and a Scanning Electron Microscope (VE-9800, Keyence Co., Ltd. Japan) were used for optical studies. Adobe Photoshop CS5 software was used to optimize brightness and contrast of images, and to compose the figures. The classification adopted here is based on previous taxonomic literatures [7], [19], [21]. Size measurements are restricted to complete or almost complete rostra without deformation. All necessary permits were obtained for the described study, which compiled with all relevant regulations. Permits of fieldwork were issued by the Board of Education, Minamisanriku Town.

The electronic edition of this article conforms to the requirements of the amended International Code of Zoological Nomenclature, and hence the new names contained herein are available under that Code from the electronic edition of this article. This published work and the nomenclatural acts it contains have been registered in ZooBank, the online registration system for the ICZN. The ZooBank LSIDs (Life Science Identifiers) can be resolved and the associated information viewed through any standard web browser by appending the LSID to the prefix "http://zoobank.org/". The LSID for this publication is: urn:lsid:zoobank.org:pub:DDBB8490-9718-4980-B6A4-5665F72B81B7.The electronic edition of this work was published in a journal with an ISSN, and has been archived and is available from the following digital repositories: PubMed Central, LOCKSS

## References

[1] Mutterlose J (1998) The Barremian–Aptian turnover of biota in northwestern Europe: evidence from belemnites. Palaeogeogr Palaeoclimatol Palaeoecol 144: 161–173 10.1016/S0031-0182(98)00081-9

[2] Christensen WK (2002) Palaeobiology, phylogeny and palaeobiogeography of belemnoids and related coleoids. Berl Palaobiologische Abh 1: 18–21.

[3] Iba Y, Mutterlose J, Tanabe K, Sano S, Misaki A, et al (2011) Belemnite extinction and the origin of modern cephalopods 35 m.y. prior to the Cretaceous–Paleogene event. Geology 39: 483–486 10.1130/G31724.1

[4] Doyle P (1993) Mollusca: Cephalopoda (Coleoidea). In: Benton MJ, editor. The Fossil Record 2. London: Chapman and Hall. pp. 229–236.

[5] Schlegelmilch R (1988) Die Belemniten des süddeutschen Jura. Stuttgart: Gustav Fischer. 149 p.

[6] Kostak M (2004) Cenomanian through the lowermost Coniacian Belemnitellidae Pavlow (Belemnitida, Coleoidea) of the East European Province. Geoline 18: 59–109.

[7] Weis R, Mariotti N, Riegraf W (2012) The belemnite family Holcobelidae (Coleoidea) in the European Jurassic: systematics, biostratigraphy, palaeobiogeography and evolutionary trends. Palaeodiversity 5: 13–49.

[8] Doyle P (1994) Aspects of the distribution of Early Jurassic belemnites. Palaeopelagos Spec Pub 1: 109–120.

[9] Iba Y, Sano S, Mutterlose J, Kondo Y (2012) Belemnites originated in the Triassic–A new look at an old group. Geology 40: 911–914 10.1130/G33402.1

[10] Sato T (1956) Corrélation du Jurassique inférieur japonais en basant sur les Ammonites Fossiles. Jour Geol Soc Japan 62: 490–503.

[11] Takahashi H (1969) Stratigraphy and ammonite fauna of the Jurassic System of the southern Kitakami massif, northeast Honshu, Japan. Sci Rep Tohoku Univ 2nd Ser 41: 1–93.

[12] Sato T, Westerman GEG (1991) Japan and South-East Asia. In: Westerman GEG, Riccardi AC, editors. Jurassic taxa ranges and correlation charts for the circum Pacific. Newsletters on Stratigraphy 24: pp. 81–108.

[13] Zittel KAV (1895) Grundzüge der Palaeontologie (Palaeozoologie). München, Leipzig, R. Oldenburg. 971 p.

[14] Doyle P (1991) The British Toarcian (Lower Jurassic) belemnites Part 2. Monogr Palaeontol Soc 145: 50–79.

[15] Doyle P, Donovan DT, Nixon M (1994) Phylogeny and systematics of the Coleoidea. Univ Kansas Paleontol Contrib 5: 1–15.

[16] Riegraf W (1980) Revision der Belemniten des Schwäbischen Jura, Teil 7. Palaeontographica 169: 128–208.

[17] Doyle P, Kelly SRA (1988) The Jurassic and Cretaceous belemnites of Kong Karls Land, Svalbard. Skrifter Norsk Polarinstitutt 189: 1–77.

[18] Weis R, Mariotti N (2007) A belemnite fauna from the Aalenian–Bajocian boundary beds of the Grand Duchy of Luxembourg (NE Paris Basin). Boll Soc Paleontol I 24: 181–184.

[19] Dzyuba OS (2011) Subfamily classification within Cylindroteuthididae (Belemnitida). Novosti Paleontol Stratigr 52: 103–107.

[20] Sachs VN, Nalnjaeva TI (1975) The Early and Middle Jurassic belemnites of the north of the USSR: Megateuthinae and Pseudodicoelitinae. Trudy Instituta Geologiya i Geofizika Sibirskoye Otdeleniye Akademiya Nauka SSSR 239: 1–192.

[21] Jeletzky JA (1966) Comparative morphology, phylogeny, and classification of fossil Coleoidea. Univ Kansas Paleontol Contrib, Mollusca 7: 1–162.

[22] Riegraf W, Janssen N, Schmitt-Riegraf C (1998) Cephalopoda dibranchiate fossils (Coleoidea) II. In: Westphal F, editor. Fossilium catalogus animalia, Pars 135. Leiden. Backhuys Publishers. pp. 5–512.

[23] Weis R, Delsate D (2006) The earliest belemnites: New records from the Hettangian of Belgium and Luxembourg. Acta Universitatis Carolinae Geologica 24: 181–184.

[24] Zhu KY, Bian Z (1984) Sinobelemnitidae, a new family of Belemnitida from the Upper Triassic of Longmenshan, Sichuan. Acta Palaeontol Sinica 23: 300–319.

[25] Chen T (1982) Mesozoic Coleoidea fauna from Xizang. Palaeontology of Xizang 4: 282–325.

[26] Stevens GR (1965) The Jurassic and Cretaceous belemnites of New Zealand and a review of the Jurassic and Cretaceous belemnites of the Indo-Pacific region. New Zealand Geol Sur Paleontol Bull 36: 1–281.

[27] Jeletzky JA (1980) Dicoelitid belemnites from the Toarcian-middle Bajocian of western and Arctic Canada. Geol Sur Canada Bull 338: 59–109.

[28] Wu S (1982) Characteristics of Early Jurassic-Early Cretaceous belemnoid assemblages from southern Xizang (Tibet). Contribution to the Geology of the Qinghai-Xizang Tibet Plateau 10: 113–121.

[29] Shiino Y, Suzuki Y, Kobayashi F (2011) Sedimentary history with biotic reaction in the Middle Permian shelly sequence of the Southern Kitakami Massif, Japan. Island Arc 20: 203–220 10.1111/j.1440-1738.2011.00760.x

[30] Blakey R (2010) Mollewide plate tectonic maps (Early Jurassic 200Ma). Available: http://cpgeosystems.com/200moll.jpg.

